# Medical Literature

**Published:** 1875-04

**Authors:** 


### Medical Literature

All of our well known medical publishers are more or less busy in the production of medical works of one kind or another. As many of our readers do not have ready access to their catalogues we will give a brief list of some of the more important works about to be published. Lindsay & Blakiston, of Philadelphia, are preparing in connection with the Messers Churchill of London, among others, the following: Clinical Studies, by Sir John Rose Cormack. The Microscope in Medicine, (4th Ed.,) by Lionel S. Beal, M. B. Experimental investigations in the action of Medicine, by T. Lauder Brunton, M. D. Diseases of the Throat and Nose, by Morell Mackenzie. A Practical Treatise on Diseases of the Eye, by Haynes Walton, and The Surgery of the Female Pelvic Organs, by Henry Savage, M. D.

Messrs Wm. Wood & Co., of New York, are busy at work on Ziemssen's Cyclopædia, the third Volume of which is announced for May. They also announce for October a translation of Uhle & Wagner's Manual of General Pathology. They have in preparation other works, the titles of which are not yet announced. Henry C. Lea, of Philadelphia, will shortly publish A Manual of Diet in Health and Disease, and a third edition of Taylor on Poisons in Relation to Medical Jurisprudence and Medicine.

Messrs. Putnam's Sons, of New York, are issuing a Series of American Clinical Lectures, which we have noticed previously. Three of the series have been published, they are by Prof's Sayer, Jacobi and Flint. Those already published are ably prepared and are presented in a neat and convenient pamphlet form, we wish Dr. Seguin and the publishers success in their enterprise.

Appleton & Co. have in press Acne; Its Pathology, Etiology, Prognosis and Treatment by L. D. Bulkley, M. D., of New York, also a Treatise on Therapeutics, by Roberts Bartholow, M. D., of Cincinnati, other works of interest will shortly be published by them.

The appearance of the second portion of the Medical and Surgical History of the War is looked for with interest, and we learn from the last report of the Surgeon General that seven hundred pages have been printed and that nearly all the plates are prepared.

